# Snapshot in surgery: How do you approach this 12‐year‐old girl?

**DOI:** 10.1002/ccr3.1614

**Published:** 2018-05-29

**Authors:** Roberto Bustos, Federico Gheza, Mario Masrur

**Affiliations:** ^1^ Division of General, Minimally Invasive and Robotic Surgery Department of Surgery University of Illinois at Chicago Chicago IL USA

**Keywords:** bezoar, gastric, giant, laparoscopic, obstruction

## Abstract

This case open questions about the dimensional limit for a laparoscopic treatment of a giant bezoar. A minimally invasive option should be considered every time a gastric obstruction is suspected, particularly for psychiatric patients, for whom a short hospital stay can be greatly beneficial.

## 1 QUESTION

How do you approach this 12‐year‐old girl with nausea and vomiting?

## 2 ANSWER

**Figure 1:**
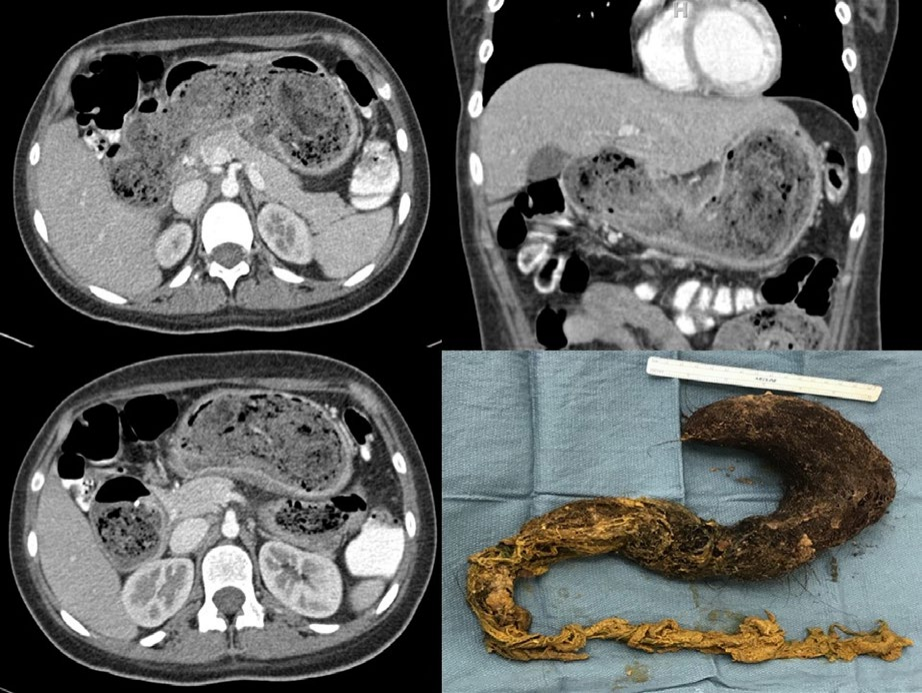
Abdominal CT scan showing distended stomach occupied by bezoar and picture of the specimen

This young lady affected by trichotillomania presented with nausea and vomiting and a CT scan was obtained (Figure 1). Small bezoars are routinely removed endoscopically. In case of huge bezoars, open surgery is the gold standard, but a laparoscopic approach can be attempted.

In this case, after the pneumoperitoneum was achieved and ports were placed, the gastrotomy was performed with Ligasure^™^ and the bezoar was taken out. The stomach was then closed. The bezoar was placed on an endobag and removed from the abdominal cavity via a 5 cm Pfannenstiel incision, covered by a wound protector. Postoperative course was uneventful and patient was discharged on postoperatory day 3. At the last follow‐up, 2 months later, the patient was asymptomatic.

## CONFLICT OF INTEREST

None declared.

## AUTHORSHIP

RB: drafted the article and data and images collection. FG: participated in the design of the work and critical revision. MM: performed surgery during the case, critical revision, and final approval.

